# A framework for healthcare interventions to address maternal morbidity

**DOI:** 10.1002/ijgo.12469

**Published:** 2018-05-23

**Authors:** Tabassum Firoz, Affette McCaw‐Binns, Veronique Filippi, Laura A. Magee, Maria L. Costa, Jose G. Cecatti, Maria Barreix, Richard Adanu, Doris Chou, Lale Say, the members of the WHO Maternal Morbidity Working Group (MMWG), Kelli Barbour, Sara Cottler, Olubukola Fawole, Luis Gadama, Atf Ghérissi, Gill Gyte, Michelle Hindin, Anoma Jayathilaka, Amanda Kalamar, Yacouba Kone, Nenad Kostanjsek, Isabelle Lange, Arvind Mathur, Mark Morgan, Stephen Munjanja, Gathari N. Gichuhi, Max Petzold, Elizabeth Sullivan, Frank Taulo, Özge Tunçalp, Rachel Vanderkruik, Peter von Dadelszen

**Affiliations:** ^1^ Department of Medicine Brown University Providence RI USA; ^2^ Department of Community Health and Psychiatry University of the West Indies Mona, Kingston Jamaica; ^3^ Department of Infectious Disease Epidemiology London School of Hygiene and Tropical Medicine London UK; ^4^ Department of Women's Health King's College London London UK; ^5^ Department of Obstetrics and Gynecology University of Campinas São Paulo Brazil; ^6^ UNDP–UNFPA–UNICEF–WHO–World Bank Special Programme of Research Development and Research Training in Human Reproduction (HRP) Department of Reproductive Health and Research WHO Geneva Switzerland; ^7^ School of Public Health Department of Population, Family and Reproductive Health University of Ghana Accra Ghana

**Keywords:** Healthcare interventions, Maternal morbidity, Noncommunicable diseases, Pregnancy complications

## Abstract

The maternal health agenda is undergoing a paradigm shift from preventing maternal deaths to promoting women's health and wellness. A critical focus of this trajectory includes addressing maternal morbidity and the increasing burden of chronic and noncommunicable diseases (NCD) among pregnant women. The WHO convened the Maternal Morbidity Working Group (MMWG) to improve the scientific basis for defining, measuring, and monitoring maternal morbidity. Based on the MMWG's work, we propose paradigms for conceptualizing maternal health and related interventions, and call for greater integration between maternal health and NCD programs. This integration can be synergistic, given the links between chronic conditions, morbidity in pregnancy, and long‐term health. Pregnancy should be viewed as a window of opportunity into the current and future health of women, and offers critical entry points for women who may otherwise not seek or have access to care for chronic conditions. Maternal health services should move beyond the focus on emergency obstetric care, to a broader approach that encompasses preventive and early interventions, and integration with existing services. Health systems need to respond by prioritizing funding for developing integrated health programs, and workforce strengthening. The MMWG's efforts have highlighted the changing landscape of maternal health, and the need to expand the narrow focus of maternal health, moving beyond *surviving* to *thriving*.

## Abstract 2

An outline of the impact of the paradigm shift from preventing maternal deaths to addressing maternal morbidity on healthcare interventions, integrating care for chronic conditions in pregnancy.

## 1 INTRODUCTION

The sustained focus on maternal health in recent years has resulted in significant progress in improving maternal health, particularly with the reduction of maternal deaths worldwide.^1^

While more work remains, countries now need to go beyond survival, with a view to establishing integrated healthcare services that can maximize the health, well‐being, and potential of women throughout their lives.^2^ This broader view of maternal health is timely, considering the changes in maternal health epidemiology and health systems ongoing in many countries. Recent years have seen many lower‐income countries move through the “obstetric transition,” a gradual shift from a pattern of high maternal mortality to low maternal mortality, and from a predominance of direct obstetric causes of maternal mortality to an increasing proportion of indirect causes, noncommunicable causes, and aging of the maternal population.^3^ However, the projected increase in the prevalence of noncommunicable diseases (NCDs)—as well as their risk factors,^4^ particularly obesity—may reverse this pattern, as the risk of death from direct causes may increase again from causes such as superimposed pre‐eclampsia and thromboembolism.^5^

The WHO convened the Maternal Morbidity Working Group (MMWG) for an in‐depth, evidence‐based approach to addressing maternal morbidity, focusing on developing standardized definitions, identification criteria, and measurement tools for the continuum of maternal morbidities. The iterative 5‐year process, results of the pilot study, and subsequent conceptual framework—described in other articles in this Supplement—have important implications for healthcare interventions and programs.

The pilot study, a cross‐sectional study of 1490 women in antenatal care (ANC) and postpartum care (PPC) conducted in Jamaica, Kenya, and Malawi, found that indirect or underlying medical conditions made up a significant proportion of clinical diagnoses in both ANC (18.0%) and PPC (8.6%) women.^6^ Of these women, 12.8% (ANC) and 11.0% (PPC) self‐reported exposure to violence. The pilot also demonstrated that women often have negative pregnancy experiences, including feelings or conditions that, while not pathologic, can persist and affect their quality of life.

**Figure 1:**
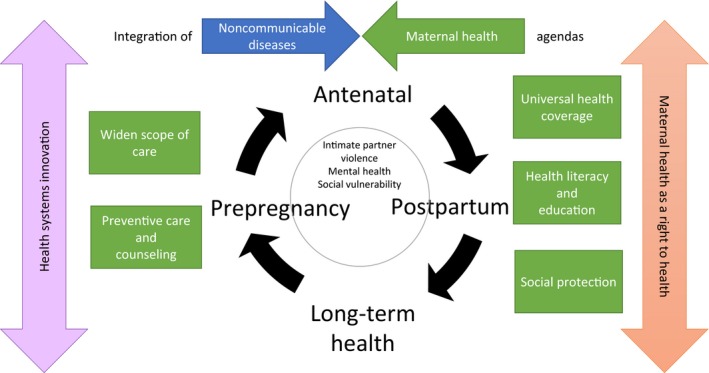
Framework for healthcare interventions to address maternal morbidity. [Colour figure can be viewed at http://www.wileyonlinelibrary.com]

To address the current complex changes in global maternal health, a rethinking of the maternal health agenda is needed.^7^ Based on the results of the pilot study and using the principles of the Strategies toward Ending Preventable Maternal Mortality (EPMM),^8^, ^9^ we explore paradigms and interventions for conceptualizing, delivering, and, ultimately, improving maternal health (Fig. 1).

## 2 SHIFTING PARADIGMS

### 2.1 Pregnancy as a window of opportunity

In recent years, there has been a movement toward viewing pregnancy as a *window of opportunity* to improve the overall health of women, whereby good maternal health care can ensure health benefits for the index pregnancy, future pregnancies, and long‐term health and well‐being of the woman. Conceptualizing pregnancy in this way allows us to situate maternal health within the life course, and offers critical entry points for women to access healthcare services in a continuous, integrated fashion, ranging from family planning to prepregnancy care, through pregnancy, labor, childbirth and the postpartum period, and continued NCD and reproductive health care.

The uniqueness of pregnancy as an event in the life course of a woman is that the physiologic demands of pregnancy act as a “stress test”.^10^ Pregnancy can reveal underlying or undiagnosed diseases that might have been dormant or unrecognized, as well as the risk of future chronic conditions.^11^ As an example, hypertensive disorders were among the most common diagnoses in both ANC and PPC women (2.9% and 4.1%, respectively) participating in the pilot study.^6^ It is well established that hypertensive disorders of pregnancy, particularly pre‐eclampsia, increase the future risk of hypertension, stroke, cardiovascular disease, and premature death.^12^ Pregnancy offers an opportunity to identify these women and undertake interventions for cardiovascular risk reduction over the longer term. Dedicated postpartum health clinics are now common in countries such as Canada, where women are offered standardized postpartum follow‐up for cardiovascular disease risk screening, and receive counseling during pregnancy.^13^

Up to 80% of the NCD burden can be prevented by addressing common risk factors.^14^ Pregnancy is a window of opportunity when women are highly motivated to make behavioral changes, which can catalyze health promotion interventions to address NCD risk factors such as smoking, unhealthy diets, and physical inactivity. A recent review showed, for instance, that providing psychosocial interventions during pregnancy can be effective for smoking cessation.^15^ In addition to improving the woman's own health, addressing NCD risk factors during pregnancy also offers a window of opportunity for intergenerational prevention of chronic diseases.^14^ Antenatal and early‐life development through epigenetic programming influences the risks of NCDs in later life. The mother's body composition, and nutritional and metabolic status during pregnancy, determine the fetal environment.^14^ Excessive pregnancy weight gain, maternal obesity, and gestational diabetes mellitus (GDM) are among some of the common cues that can cause epigenetic changes, and result in multigenerational cycles of disease.^14^ These can be addressed or screened for during pregnancy.

### 2.2 Merging the maternal health and NCDs agendas

Maternal health care has traditionally focused on diagnosing and managing obstetric complications. Yet our pilot study found that medical problems had similar levels of prevalence as obstetric ones in antenatal women, and accounted for the majority of postpartum diagnoses, especially in Jamaica and Kenya.^6^ Maternal health is intimately and reciprocally linked to NCDs. Pre‐existing conditions (such as anemia and valvular heart disease) increase both maternal morbidity and mortality.^16^, ^17^ Conversely, complications of pregnancy can increase the prevalence of chronic health conditions, influencing not only future pregnancies but also the long‐term health of women. Common examples include pre‐eclampsia (discussed above) and GDM, which increases the risk of future type 2 diabetes, and can be further influenced by ethnicity.^18^, ^19^

Combined NCDs are the leading cause of death in women worldwide, accounting for 65% of all deaths.^20^ Estimates suggest that, in 2030, a two‐thirds reduction in maternal and child deaths, and a one‐third reduction in NCD mortality would mean 210 000 fewer maternal deaths and 690 000 fewer NCD deaths in women and girls aged 5–49 years, and 2.4 million fewer NCD deaths in women aged 50–69 years.^21^ In many settings, maternal and reproductive health services are the only potential points of contact for diagnosing and preventing NCDs. Efforts toward addressing NCDs have often, however, been underfunded and misdirected, and have not prioritized women of reproductive age. The maternal and NCD agendas can no longer be approached separately; rather, they should intersect and be synergistic. This is reflected in the United Nations Secretary‐General's Global Strategy for Women's, Children's and Adolescents’ Health (2016–2030),^22^ which recommends that health care for NCDs be provided as part of an integrated approach to promote women's and children's health.

A paradigm shift is also required within our conceptualization of NCDs. Some have argued that the term “noncommunicable” is a misnomer and misleading, a problem that is exacerbated by the implication that individual (rather than societal) factors are the key determinants.^23^ This has led to a reframing of these conditions as “socially transmitted conditions” rather than “noncommunicable”.^23^ While there are individual physiologic or lifestyle choices that are at play, NCDs are also affected by broader, population‐level risk factors (such as economic, social, and environmental factors: urbanization, globalization, poverty, inequity). This is reflected in an analysis within the Global Burden of Disease study,^24^ which found that the interplay of behavioral, environmental, occupational, and metabolic risk factors can explain half of global mortality, and more than one‐third of global disability‐adjusted life years (DALYs). Women are thus particularly vulnerable to NCDs, given their lower societal and economic status in many countries. They are also disadvantaged when it comes to prevention and access to care while bearing social, sexual, psychologic, and financial stigmatization when they are affected by NCDs.^25^

### 2.3 Widening the scope of maternal health services

Historically, expanding the provision of emergency obstetric care has been one of the cornerstones of global efforts to prevent maternal mortality. Although important, given the diversity of underlying factors contributing to maternal mortality, it has been recognized that focusing solely on emergency obstetric care may not be the first (or only) priority in all settings.^26^ The scope of maternal health services should reflect the patterns of maternal health conditions women experience. The need for a deeper understanding of the role of maternal morbidity in women's health includes a greater appreciation that these conditions detract from women's health and wellness, and can contribute to negative experiences of pregnancy itself.

A key finding of the pilot study is that indirect or medical conditions contributed significantly to maternal morbidity. To address this, the maternal health community needs to expand its focus beyond pregnancy and childbirth alone to include linkages to prepregnancy care and long‐term health. The sexual, reproductive, maternal, newborn, child, and adolescent health (SRMNCAH) continuum of care offers critical entry points to screen women for NCDs, and can therefore be an opportunity to provide links to preventive care and early intervention. Maternity care is also an entry point to health promotion activities, and an opportunity to deliver key information, resources, and services that can lead to healthier choices and lifestyles.

A Postpartum Think Tank meeting hosted by the US‐based Association of Maternal and Child Health Programs (AMCHP) identified that integrated services and seamless transitions from the prepregnancy through the postpartum period were a priority.^27^ Prepregnancy care should be the “third” routine component of maternal health care, and of equal importance to antenatal and postpartum care. Similarly, emphasis should be placed on strengthening postpartum services, and extending postpartum care beyond 6 weeks when necessary, and improved integration with other medical specialties. Postpregnancy care could potentially be considered as “interconception”^28^ care as well as an opportunity to promote women's optimal health. By widening the scope of maternal health services, especially to NCDs, continuity in health care, and transitioning from maternity care to primary care services, becomes critical. Coordinated and facilitated transitions between maternity care services and primary care is often lacking, which leaves women at risk of “falling through the cracks.”

## 3 TRANSLATING PARADIGMS INTO INTERVENTIONS

### 3.1 Health systems response

The changing healthcare needs of women have profound implications for health systems, and services will need to be reorganized. Health systems are ill‐equipped to keep pace with the changing patterns of disease and the need for an expanded range of services for women.^29^ Integration of services and healthcare platforms across levels of care, medical specialties, and clinical and social care is needed. Adequate referral services, especially for intimate partner violence and mental health conditions, as seen in our pilot study, are also critical for an effective response. The content and complexity of such a service package could be informed by the local context of both maternal health conditions and the health system. This will, of course, require resource allocation, prioritized funding, and political commitment.

Health workforce strengthening to respond to the care of women with medical or chronic conditions will need the dedicated training and creative diversification of the workforce, especially at the community level. Training can include more on NCDs in the curricula of all cadres of health workers such as community health workers (CHWs), midwives, nurse practitioners, and physician assistants. Content and competencies, for example, could be adapted from existing resources like the Royal College of Obstetricians and Gynaecologists (RCOG) advanced training specialist module (ATSM) on maternal medicine.^30^ Another crucial area in need of training is intimate partner violence and other sensitive topics; interviewers (trained health workers) in the pilot study felt unprepared to engage in these discussions. While the skillset of the existing workforce is being improved, effort will be needed to ensure that the basic skills curricula for these personnel are updated to include new service components and skills. Regardless of health worker cadre, these efforts should be harmonized with ongoing processes to standardize the definition and the expected competencies of skilled health personnel caring for women throughout their lives.^31^

Reorganization of healthcare services will need innovative approaches. The WHO antenatal care guidelines, for example, have suggested that group ANC may be offered as an alternative to individual ANC for pregnant women, depending on a woman's preferences, and provided that the infrastructure and resources are available.^32^ In some low‐ and middle‐income country (LMIC) settings, such as Jamaica, group health promotion activities or talks precede individual care sessions. The group ANC model can be especially useful for women with chronic conditions. A recent study has shown that women with GDM participating in group ANC are more likely than those receiving traditional ANC to complete postpartum glucose tolerance testing, have a higher rate of breastfeeding initiation, and higher rates of exclusive breastfeeding at their postpartum visit.^33^

The biggest challenge, perhaps, will be to strengthen primary care services to ensure that women have continuous access to high‐quality care services to address their health needs across the lifespan.^34^ In LMICs, however, some of these general medical services may be overcrowded, and focused on an older demographic. It may be useful to consider other care delivery options for this population, such as initially integrating services into child health clinics, and using community health centers or networks.^34^

When designing these integrated services, considerations include potential deterioration of service quality and patient satisfaction, and overburdening frontline healthcare workers. It may be useful to consider task shifting to ensure that demand is met in a cost‐effective way, while maintaining quality. A core component of Brazil's Family Health Strategy is the extensive and effective use of CHWs to deliver primary care services, including chronic disease management and health promotion in addition to supporting healthy pregnancies.^35^ CHWs are responsible for registering every family in their area, monitoring living conditions and health status, and providing primary care. CHWs are also able to resolve many low‐level problems, and communicate with physicians and nurses. Another successful example comes from the GDM program in Vida Nueva, Colombia, which engaged CHWs to raise awareness, make referrals for screening, and follow‐up after diagnosis.^36^

## 4 APPROACHES TO DELIVERING MATERNAL HEALTH SERVICES

### 4.1 Prepregnancy care

The prepregnancy period serves as a critically important entry point to influence optimal health, nutrition, and birth preparedness. Adolescents, for whom targeted interventions during this time are needed, are particularly vulnerable and are often neglected.^37^

Firstly, engaging women of reproductive age in the prepregnancy period allows the opportunity to determine fertility intentions, and therefore plan for pregnancy in a more careful and considered way, especially when women seek contraceptive services. There are no global recommendations on routine counseling regarding pregnancy intention, and a systematic review is underway to determine the effectiveness of incorporating questions of pregnancy intention into primary health care.^38^ The WHO Medical Eligibility Criteria for Contraceptive Use^39^ is a simple and pragmatic program of guidelines that summarizes the risk of specific contraceptive methods in women with specified chronic medical conditions, and can be used in most settings.

Secondly, the evidence increasingly points to earlier care before pregnancy to improve women's health as well as pregnancy outcomes for the mother and newborn.^40^, ^41^ Prepregnancy care and counseling are particularly important for women with pre‐existing medical conditions. Some medical conditions may be exacerbated by the physiologic changes of pregnancy, and close monitoring of a carefully planned pregnancy is optimal. Some very common examples of these conditions include pre‐existing diabetes, chronic hypertension, sickle cell disease, cardiomyopathy, and HIV infection. Yet, an integrative review on the prevalence of the use of prepregnancy services by women with chronic health conditions reported estimates of engagement with prepregnancy care ranging between 18.1% and 45%, with most studies focusing on women with types 1 and 2 diabetes.^42^ These estimates are likely to be lower in resource‐constrained settings, where such services may not be available. Global guidance on the prepregnancy care of women with medical conditions—similar to that available for ANC and emergency obstetric care—would be especially useful for LMICs.

### 4.2 Antenatal care

As informed by our pilot study as well as other studies on maternal morbidity suggesting that some pre‐existing conditions are diagnosed for the first time during pregnancy,^43^ a general health assessment could be included as part of maternal history‐taking. The findings of the pilot study also highlight that the focus of routine ANC could be broadened beyond the care of chronic conditions to also include a simple way of screening for intimate partner violence and mental health conditions. Prevalence of intimate partner violence may increase during pregnancy, although the data are inconsistent.^44^ A systematic review shows that screening increases the identification of women experiencing intimate partner violence, and that pregnant women in antenatal settings may be more likely to disclose intimate partner violence when screened.^45^ While screening increases identification, this review found insufficient evidence to justify screening in all healthcare settings.^45^ This highlights the importance of taking the local context into consideration.

WHO has long recognized that integration of antenatal care with other health services is a key strategy to reduce missed opportunities for patient contact, and to effectively address the comprehensive health needs of women. About 70% of pregnant women in LMICs have at least one antenatal visit, providing a crucial opportunity for providing integrated services.^46^

Antenatal care has traditionally been bundled with prevention of mother‐to‐child HIV transmission services and syphilis screening.^46^ Countries can capitalize on the relatively high ANC coverage in many LMICs by further integrating services related to chronic diseases. A successful example comes from a collaboration between the Colombian government, Accenture Development Partnerships, and the World Diabetes Foundation, and the project has raised NCD awareness and capacity building to integrate GDM care into existing antenatal services. In Colombia, this has raised the screening for GDM from 5% to 97% in 3 years.^36^ While it may make sense to integrate services with routine ANC, one study has shown that coverage for most of the specific elements of ANC is generally low.^47^

### 4.3 Postpartum care

The postpartum period can be seen as the “fourth trimester,” and has been described as a “critical transition period with unmet maternal health needs”.^48^ Similar to the findings of our pilot study, other studies have found that postpartum women struggle with a variety of new health problems in the immediate postpartum period.^49^ Postpartum women have expressed that the range of experiences they encounter is not often fully acknowledged, and that clinical interactions are often limited to obstetric observations. The results of the pilot study suggest that postpartum follow‐up is particularly important to address symptoms and conditions that women identify as causing significant discomfort and impact their functioning.

Postpartum care may be more comprehensive if concerns addressed include issues around physical recovery from childbirth, sleep and fatigue, sexuality, contraception and birth spacing, childcare challenges, and mood and emotional well‐being.^48^ To make this more efficient, it may be helpful to develop checklists.

The EPMM report recommends the integration of maternal and newborn health services, capitalizing on women's presence at a healthcare facility to address the needs of both her and her infant.^8^ This strategy can be especially important in the postpartum care of women with chronic conditions as it could provide an opportunity to address the continuity of care needs as well as increase the uptake of family planning services. With the competing demands of motherhood and family responsibilities, women may be more likely to attend and engage in postpartum appointments when they are delivered with existing services such as newborn services. Synchronized scheduling of medical and child health clinics could be a starting point, so that these services are offered on the same day, and allow women efficient access to both types of care. Delivering lifestyle interventions such as postpartum weight loss and smoking cessation along with routine health maintenance is also most likely to be successful if delivered with newborn care.

Group care for the mother–baby pair is another way to integrate maternal and newborn healthcare services. The Centering Parenting model offers group care that includes well‐child health assessments, immunizations, and developmental screenings along with maternal health and wellness services and education, including: postpartum care, family planning, mental health, breastfeeding, oral health, relationships, reproductive health, infant attachment, life balance, and weight goals.^50^ Many participants considered attention to maternal wellness a benefit of the model, as the competing demands of children and families were identified as a barrier for women to seek care for their own postpartum health.^50^

The postpartum visit also offers an opportunity to screen for mental health conditions, such as postpartum depression. The WHO recommendations for the postpartum care of women highlight psychosocial support by a trained person for the prevention of postpartum depression among women at high risk of developing this condition.^51^ Psychosocial and psychological interventions have been shown to significantly reduce the number of women at risk who go on to develop postpartum depression.^52^ Interventions include the provision of intensive, professional postpartum home visits, telephone‐based peer support, and interpersonal psychotherapy.^52^ Counseling and interventions are also needed to address issues such as a nonsupportive partner, or families coping with the impact or outcomes of pregnancy.

### 4.4 Taking a rights‐based approach

By taking a rights‐based approach to health and well‐being, women can attain their full potential. The Global Strategy for Women's, Children's and Adolescents’ Health^2^—a roadmap for achieving the highest standard for health—envisions that women and children will not only survive, but thrive. This requires a comprehensive approach that also includes more distal, nonclinical risk factors, or social determinants of health. These factors not only create social vulnerabilities but also influence health‐seeking behavior and access to care, necessitating a rights‐based approach to maternal health—a guiding principle of the EPMM strategies. The maternal health community needs to explore strategies for addressing the social vulnerability of women and children, which can be especially acute for young, unemployed, or unmarried women, or women in abusive relationships. These families are at high risk of intimate partner violence, anxiety and depression, malnutrition, and limited healthcare access due to resource restrictions.

Social protection aims to provide a minimum level of subsistence for all women, and can extend benefits to marginalized groups.^53^ Social protection can improve education and nutritional status, empower women, benefit their health, and support them as caregivers.^53^ Jamaica, for example, has the Programme of Advancement Through Health and Education (PATH), which provides economic and nutritional support to families screened to be in need.^54^ Social protection may mean offering complex interventions, depending on the scenario. In the case of intimate partner violence, these may entail skills training to enable independent income generation, counseling, temporary housing, and permanent rehousing.

Transformative social protection measures such as legislative, regulatory, and policy measures are needed.^53^ Job security, appropriate work conditions, and maternity leave (especially paid maternity leave) are important for women who have suffered pregnancy‐related morbidity. A rights‐based approach also includes the empowerment of women through health literacy, as women will be better able to access health resources, understand counseling, and participate in and make informed decisions about health planning, subsequently enhancing maternal and child outcomes.

Universal health coverage (UHC) is an essential component of social protection. Women with chronic conditions undoubtedly experience financial hardship due to the cost of health care. Many LMICs such as Ethiopia, India, and Rwanda have achieved UHC.^53^ The recently appointed Director‐General of WHO, Dr Tedros Ghebreyesus, has highlighted that UHC will allow access to both preventive and curative care.^55^ Achieving UHC for maternity care is a priority recommendation in the EPMM strategies, and a core indicator identified for achieving progress toward this priority is coverage of essential services through an “essential covered SRMNCAH services package.” This includes a cost‐effective, priority‐covered package of essential services and commodities for prepregnancy, ANC, labor and delivery, PPC, and family planning.^8^ The EPMM report also recommends that governments institute publicly funded insurance to protect women and their families from out‐of‐pocket costs, as well as the expansion of services through progressive mandatory prepayment and pooling of funds, with exemptions for the poor.^8^ Brazil has an innovative public service through which health centers receive financial incentives if registered pregnant women receive the minimum package for ANC, including a first visit during the first trimester, at least six visits during pregnancy, completion of recommended lab tests, and at least one visit during the postpartum period (all checked electronically through an information system).^56^

A broader approach to maternal health that addresses contemporary challenges and patterns of disease is needed. We propose a framework to meet this challenge that goes beyond traditional maternity care models where women are in contact with healthcare systems only through pregnancy to 6 weeks postpartum. Maternal health service delivery can take a broader life‐course perspective, including prepregnancy care, longer‐term health care, and improved integration with existing health programs and services. Rather than being in silos, the maternal health and NCD agendas should be synergistic; interventions are needed at the points of intersection. The maternal health community should look beyond survival, to wellness and attainment of the highest achievable level of health for all women. This reflects women's full value as contributing members of families, communities, societies, and economies.

## AUTHOR CONTRIBUTIONS

TF led the manuscript writing process. AMB, VF, LAM, MLC, DC, MB, JGC, and RA contributed to and edited the manuscript. LS conceptualized the maternal morbidity measurement initiative. All authors read and approved the final manuscript.

## MMWG MEMBERS

Kelli Barbour, Sara Cottler, Olubukola Fawole, Luis Gadama, Atf Ghérissi, Gill Gyte, Michelle Hindin, Anoma Jayathilaka, Amanda Kalamar, Yacouba Kone, Nenad Kostanjsek, Isabelle Lange, Arvind Mathur, Mark Morgan, Stephen Munjanja, Gathari N. Gichuhi, Max Petzold, Elizabeth Sullivan, Frank Taulo, Özge Tunçalp, Rachel Vanderkruik, Peter von Dadelszen.

## CONFLICTS OF INTEREST

RA is the Editor of the *International Journal of Gynecology and Obstetrics* but was not involved in the peer review or approval of the manuscript for this supplement. The authors declare no conflicts of interest.
